# Antimicrobial Testing of *Schinus molle* (L.) Leaf Extracts and Fractions Followed by GC-MS Investigation of Biological Active Fractions

**DOI:** 10.3390/molecules25081977

**Published:** 2020-04-23

**Authors:** Giovanni Turchetti, Stefania Garzoli, Valentina Laghezza Masci, Carla Sabia, Ramona Iseppi, Pierluigi Giacomello, Antonio Tiezzi, Elisa Ovidi

**Affiliations:** 1 Department for the Innovation in Biological, Agrofood and Forestal Systems, Tuscia University, 01100 Viterbo, Italy; g.turchetti@unitus.it (G.T.); laghezzamasci@unitus.it (V.L.M.); antoniot@unitus.it (A.T.); eovidi@unitus.it (E.O.); 2 Department of Drug Chemistry and Technology, Sapienza University, 00185 Rome, Italy; pierluigi.giacomello@uniroma1.it; 3 Department of Life Sciences, University of Modena and Reggio Emilia, 41121 Modena, Italy; carla.sabia@unimore.it (C.S.); ramona.iseppi@unimore.it (R.I.)

**Keywords:** *Anacardiaceae*, pepper tree, antimicrobial, low-pressure column chromatography, plant compounds, minimum inhibitory concentration, gas chromatography-mass spectrometry

## Abstract

*Schinus molle* (L.) is a dioecious plant of the Anacardiaceae family, originating in South America and currently widespread in many regions throughout the world. In this work leaf extracts and derived low-pressure column chromatography (LPCC) fractions of *S. molle* L. male and female plants were investigated for the antimicrobial activity. Leaf extracts were tested on microbes *Escherichia coli*, *Pseudomonas aeruginosa*, *Staphylococcus aureus*, *Enterococcus faecalis*, *Candida albicans* and *Bacillus subtilis*. Furthermore, the extracts showing antimicrobial activity were fractionated by LPCC and the obtained fractions tested on the same microorganism strains. Positive fractions were investigated by gas-chromatography/mass spectrometry (GC-MS) and were seen to be rich in sesquiterpenes, sesquiterpenoids and other terpens. The obtained effects highlighted the antimicrobial properties of *S. molle* (L.) leaf compounds and revealed their importance as a source of bioactive molecules of potential pharmaceutical interest. To our knowledge, this is the first paper reporting investigations on the chemical composition of the extracts and derived positive fractions from *Schinus molle* (L.) plants grown in central Italy

## 1. Introduction

Over the decades, the extensive use of antibiotics, especially prophylaxis, has led to the development of resistant pathogens [1]. Disease control to combat emerging and re-emerging pathogen resistance can be counteracted by modifying existing antibiotics [2] and searching for new antibiotics from natural products, which can provide a range of molecules to be tested for this purpose.

From this perspective, the literature of the last 20 years has grown in quantity and quality and many studies have been carried out to test the antimicrobial activity of extracts from plant matrices. Gomes and colleagues evaluated antibacterial activity against multidrug-resistant strains of hospital origin and standard strains using extracts exploiting a plant matrix of waste from the Brazilian pepper tree (*Schinus terebinthifolia* Raddi) processing industry chain [3]. Sharma et al. evaluated the potential antibacterial and antibiofilm capacity of bioactive compounds extracted from onions at different stages of age on gram-negative *Escherichia coli* and *Pseudomonas aeruginosa* and gram-positive *Staphylococcus aureus* and *Bacillus cereus* [4]. Several studies have shown the antibacterial activity of extracts from plants such as essential oils [5,6,7,8,9]. Industrial hemp was used to obtain essential oil rich in terpenic compounds and this oil was tested against pathogenic and spoilage microorganisms to evaluate the antimicrobial activity [10]. Lavandin essential oil (liquid and vapour phase) showed an antimicrobial activity against gram-negative (*E. coli*, *Acinetobacter bohemicus*, and *Pseudomonas fluorescens*) and gram-positive (*B. cereus* and *Kocuria marina*) bacteria [11].

*S. molle* (L.), known as “false pepper” or “pink pepper” due to the edible red/pink fruits whose flavour and fragrance are reminiscent of *Piper nigrum* (L.), is an evergreen plant belonging to the Anacardiaceae family. It is native to South America where it is known as the Peruvian pepper tree [12], whereas it is distributed worldwide as an ornamental plant. Many papers attribute some pharmacological proprieties to the pepper tree, such as antiproliferative [6,13,14,15,16], antioxidant [9,14], antifungal [17,18], hypotensive [19], anti-inflammatory [20], and analgesic [21] effects, as well as antimalarial [22], acaricidal [23,24], repellent and insecticidal properties [25,26,27,28].

Antibacterial activity has been investigated in extracts and essential oils from leaves, flowers, berries, and bark derived from this plant [5,6,7,8,9,18,28,29,30,31,32].

In our search for new compounds of possible pharmaceutical interest, we previously carried out chemical investigations of male and female *S. molle* (L.) fresh leaf extracts and they were shown to be mainly composed of sesquiterpene hydrocarbons and monoterpene hydrocarbons [33]. We found the presence of molecules—such as elemol, β-elemene, β–caryophyllene, germacrene D, bicyclogermacrene, spathulenol, α-eudesmol, β-eudesmol, γ-eudesmol, isocalamendiol, sabinene and n-hexadecanoic acid—that could be related to possible antibacterial, antiviral and antifungal activities, as already reported in literature [34,35,36,37,38,39,40,41,42,43,44,45].

For the first time, in this work the antibacterial activity of extracts and their fractions of leaves from *S. molle* (L.) grown in central Italy was evaluated on clinically relevant bacterial strains and the chemical composition of biological active fractions investigated.

## 2. Results

The investigation of antibacterial activity was carried out on female and male leaf extracts of *S. molle* (L.) using four solvents with increasing polarity with this crescent order: petroleum ether, diethyl ether, acetone and distilled water.

### 2.1. Testing of Antimicrobial Activity

The antibacterial assay of the *S. molle* (L.) leaf extracts was carried out using the disk diffusion test. Treatments with petroleum ether (SM♀L1) and diethyl ether (SM♀L2) on extracts from the *S. molle* (L.) female plant (Table 1) and with petroleum ether (SM♂L1) and diethyl ether (SM♂L2) on the *S. molle* (L.) male plant (Table 2) showed inhibition zones between 8 and 17 mm on *Staphylococcus aureus*, *Enterococcus faecalis*, *Candida albicans* and *Bacillus subtilis*. A minimal inhibitory concentration (MIC) assay of the tested extracts with a positive response in the disk diffusion test (DDT) recorded values between 25 µg/mL and 400 µg/mL. The DTT of the leaf extracts from the female *S. molle* (L.)—SM♀L1, SM♀L2, SM♀L3 (acetone extract), SM♀L4 (water extract)—and the male—SM♂L1, SM♂L2, SM♂L3, SM♂L4—produced no effects on *E. coli* and *P. aeruginosa*. The extracts SM♀L3, SM♀L4, SM♂L3 and SM♂L4 had no effects on any of the strains used (Table 1 and Table 2).

The extracts with antimicrobial activity (SM♀L1, L2 and SM♂L1, L2) were fractioned by the low-pressure column chromatography (LPCC) technique using solvents with increasing polarity in this order: petroleum ether 100%, petroleum ether/ethyl acetate 95%/5%, 90%/10%, 80%/20%, 70%/30% and 0%/100% and methanol 100%. The obtained fractions have been investigated by disk diffusion and microdilution tests. Concerning the size of the inhibition halo and the MIC values, as shown in Table 1 and Table 2, an increase in the antibacterial activity occurred for many of the tested fractions.

### 2.2. Chemical Investigation of Positive Fractions

The fractions derived from female and male *S. molle* (L.) leaf extracts that produced an inhibition halo equal to or greater than 10 mm were further investigated with the gas-chromatography/mass spectrometry. The identification of the components separated by GC/MS was carried out by comparing the mass spectra for each compound with that reported in mass spectrometry (MS) libraries and by calculating their linear retention indices (LRIs). Table 3 shows the chemical composition of petroleum ether and diethyl ether fractions from female *S. molle* (L.) leaves; 40 identified molecules are reported. Table 4 describes the chemical composition of petroleum ether and diethyl ether fractions from male *S. molle* (L.) leaves; 12 identified molecules are listed. The composition of the biologically active fractions obtained by LPCC from the male and female extracts in petroleum ether showed a particular abundance of sesquiterpenes and monoterpene hydrocarbons. The fractions obtained from the male and female diethyl ether extracts were rich in sesquiterpenes and alcohol terpenes; fatty acids, phenols, esters and hydrocarbons were also present.

## 3. Discussion

Plant secondary metabolites possess numerous biological activities and are useful in the treatment of human and animal health problems. Antibacterial activities have been well defined in different classes of natural compounds and, due to the high incidence of antibiotic resistance, the search for new antibiotics is ongoing.

The antibacterial activity of the SM♀L1-F7 fraction against *S. aureus* showed an increase in the inhibition halo, from 10.3 ± 1.5 mm to 20 ± 1 mm, and a reduction in the MIC, from 400 µg/mL to 128 µg/mL, compared to the extract SM♀L1. The biological activity could be related to the presence of β-eudesmol (67.9%), together with cyclotetradecene (15.7%), γ- and α-eudesmol (6.4% and 3.9%, respectively), dehydroxyisocalamendiol (3.4%) and elemol (2.7%). Salem and colleagues [31] demonstrated the antimicrobial activity of the water extract of wood branches of *S. molle* (L.), which has a high percentage of β-eudesmol; this work supports the relationship between the chemical content found and the biological activity observed. Furthermore, the lowest MIC against *S. aureus* was obtained in the fraction SM♀L1-F4, containing elemol (38.1%), β-terpinene (33.8%)—known for its antibacterial activity against *S. aureus* [6], β-eudesmol (10.2%), dehydroxyisocalamendiol (8.5%) and γ- and α-eudesmol (5.2% and 4,1%, respectively). The effects observed against against *S. aureus* led us to consider a synergism between the molecules present in the fraction and the antimicrobial activity of this strain.

The fraction SM♀L1-F5 showed good inhibitory activity against *B. subtilis,* with an increase in halo diameter from 14.3 ± 1.2 mm to 24.7 ± 0.6 mm for the disk diffusion test and an MIC reduced from 400 µg/mL to 8 µg/mL compared to extract SM♀L1. Similar values were also obtained from the fraction SM♀L1-F6 (DDT = 21.7 ± 1.2 mm, MIC = 8 µg/mL). The major constituent of these fractions was elemol (SM♀L1-F5 = 74.6% and SM♀L1-F6 = 71.6%). In the fraction SM♀L1-F5 α-, β- and γ-eudesmol (9.4%, 8.0% and 4.3%, respectively) were present; these molecules are known for their antimicrobial effects [31,36]. In different quantities (α- = 3.9%, β- = 17.4% and γ-eudesmol = 4.2%), these were also found in the fraction SM♀L1-F6.

The results of the SM♀L2-F4 fraction against *S. aureus* showed an increase in antibacterial activity compared to the extract SM♀L2. The inhibition halo increased from 10 mm to 18 mm, while the MIC reduced from 400 µg/mL to 64 µg/mL. In this fraction, cyclotetradecene (77.6%) and 2,4-di-*tert*-butyl phenol (2,4 DTBP) (22.4%) were identified. In particular, 2,4 DTBP is known to be an antifungal and cytotoxic molecule, as has been widely documented [46,47,48]. Aissaoui et al. [49] also described the antibacterial potential of this compound.

Among fractions obtained from the extract SM♀L2, only SM♀L2-F4 showed a modest activity against *C. albicans*. A reduction in its inhibition halo was observed, from 14.3 ± 2.1 mm to 9.7 ± 0.6 mm, and a decrease was seen in the MIC from 50 µg/mL to 32 µg/mL.

Regarding *B. subtilis*, fractionation produced better effects. The two fractions SM♀L2-F2 and SM♀L2-F3 were active, with inhibition halos of 25.7 ± 0.6 mm and 24.7 ± 1.2 mm and MICs of 1 µg/mL and 2 µg/mL, respectively. These values were the lowest recorded. The GC-MS analysis of SM♀L2-F2 revealed the presence of 3,7,11,15-tetramethyl-2-hexadecen-1-ol (86.9%), β-terpinene (5.8%), hexadecanoic acid (4.0%) and α-, β- and γ-eudesmol (1.0%, 1.3% and 1.0%, respectively). SM♀L2-F3 consisted of only phytol, a stereoisomer of 3,7,11,15-tetramethyl-2-hexadecen-1-ol with a similar antimicrobial activity [50]. Both of these molecules belong to the terpene family. Furthermore, regarding phytol, recent investigations have demonstrated anxiolytic, metabolism-modulating, cytotoxic, antioxidant, autophagy and apoptosis-inducing, anti-nociceptive, anti-inflammatory and immune-modulating effects [51].

The fraction SM♀L2-F4 was the only one to show activity in response to *E. faecalis,* with an increase in the inhibition halo from 8.3 ± 0.6 mm to 14.7 ± 1.2 mm and a reduction in the MIC from 200 µg/mL to 128 µg/mL.

As shown in Table 2, in the SM♂L1-F1 fraction the increase in antibacterial activity in response to *B. subtilis* was related exclusively to the disk diffusion test (not to the MIC). Indeed, the inhibition diameter reached 26.3 ± 0.6 mm, compared to 13.7 ± 0.6 mm for the SM♂L1 extract. This phenomenon could be due to the presence of molecules already known for their antibacterial activity, such as germacrene-D [41,42], δ-cadinene [52], elixene [53,54] and β-caryophyllene [39,41].

As already reported for *Piper nigrum* (L.) fruits [40], there could be relationship between the presence of isocalamendiol and the antibacterial activity against *S. aureus*. In this study, the SM♂L1-F6 fraction showed a good antibacterial activity against this strain, with a halo of 16.7 ± 0.6 mm and an MIC of 8 µg/mL. In this fraction, isocalamendiol (84.8%), β-eudesmol (12.9%) and spathulenol (2.3%) were present. The antibacterial activity of β-eudesmol has been evaluated through the analysis of essential oils of Amazonian species of the genus *Guatteriopsis* [36] and a low activity against *S. aureus* was reported, with an MIC of over 1000 µg/mL. Our results supported the hypothesis of an antibacterial activity against *S. aureus*, exerted by isocalamendiol or by a synergistic action of the three molecules. The inhibitory activity against *S. aureus* could be associated with the high presence of β-eudesmol, also found in the fractions SM♂L1-F10 (91.7%) and SM♀L1-F7 (67.9%), which have a DDT = 15.3 ± 1.2 mm/MIC = 128 µg/mL and a DDT = 20 ± 1 mm/MIC = 128 µg/mL, respectively.

Interesting effects were also recorded for SM♂L1-F7 (DDT = 15.3 ± 0.6 mm, MIC = 16 µg/mL). As revealed by GC-MS investigations, the fraction consisted of β-eudesmol (59.6%), spathulenol (14.2%), isocalamendiol (12.2%), squalene (7.0%) and isoaromadendrene epoxide (7.0%).

Some papers have reported the experimental data antibacterial activity against *S. aureus* of isoaromadendrene, epoxide [55]. The ineffectiveness of squalene was reported by Sharma et al. [56] in a study of the ethanolic extract from leaves of *Syzygium jambos L*. (Alston), in which no antibacterial activity occurred during the testing of squalene.

Most of the fractions derived from SM♂L2 exhibited inhibitory activity against the fungus *C. albicans*, mainly expressed by the fraction SM♂L2-F6, with a similar DDT result (11.7 ± 1.5 mm in the fraction compared to 12.7 ± 0.6 mm in the total extract) and enhanced MIC activity of 400 µg/mL to 64 µg/mL. As shown by GC-MS investigations, the fraction consisted of hexadecanoic acid. Biological activities of hexadecenoic acid reported in literature, such as larvicidal [57], anti-inflammatory [58] and antimicrobial [35,36]. In this work, we highlighted the antifungal and antibacterial activity of the fatty acid found in the fraction. The antibacterial activity of hexadecanoic acid was confirmed against *B. subtilis*, with an increase in the inhibition halo from 11.3 ± 1.5 mm in the total extract up to 28.3 ± 1.2 mm in the fraction SM♂L2-F6. This last fraction, in line with the cited literature, also showed an increase in antibacterial activity with the broth microdilution method, with an MIC reduced from 100 µg/mL to 16 µg/mL.

The fraction SM♂L2-F7 showed the biggest inhibition halo (29.7 ± 0.6 mm) recorded, an activity in contrast with the increase in the MIC value from 100 µg/mL to 256 µg/mL. The composition of this fraction was more heterogeneous than the fraction SM♂L2-F6 (Table 4). The components, oxalic acid [59], 1-docosene [60], β-eudesmol [31] and phenol 2,4-di-*tert*-butyl- [47,48,49], are known, in the literature, for their correlation with antimicrobial activity, confirming the inhibitory effect found in our work.

## 4. Materials and Methods

### 4.1. Reagents

All the solvents used for chemical analysis, extraction and fractionation purposes were analytical grade (Sigma-Aldrich, Darmstadt, Germany). The silica gel (60 Å, 0.04–0.063 mm) was from Macherey-Nagel (Düren, Germany). The microorganism media and reagents for antimicrobial tests were purchased from Sigma–Aldrich (Darmstadt, Germany).

### 4.2. Plant Materials and Extraction

The leaves of male and female *S. molle* (L.) plants were collected during the flowering period from the “Angelo Rambelli” botanical garden in Viterbo (Viterbo, Italy) and identified by Dr. Monica Fonck (Scientific Supervisor) with the number AS21 (male plant) and AS22 (female plant) in the Botanical Garden Catalogue. The leaves were washed with distilled water, air-dried in dark conditions at room temperature and stored at −80 °C until lyophilization. The frozen leaves were lyophilized for 3 days to eliminate traces of water, ground to obtain a powder (4 gr for female leaves and 4 gr for male leaves) and stored at 4 °C. In order to perform serial extractions, the powder was put in a cellulose thimble and processed in a soxhlet apparatus. Three solvents with increasing polarity—petroleum ether (PE), diethyl ether (DE) and acetone (AC)—were used. After the acetone extraction, the solid residue was macerated in distilled water overnight and the aqueous fraction filtered through Whatman paper. The solvent was evaporated by a rotary vapor (RV 08-VC, IKA, Staufen, Germany) and the dried residues put into glass vials and stored at 4 °C until use. The obtained extracts were named as SM♀L1 (PE extract of female leaves, 312 mg), SM♀L2 (DE extract of female leaves, 60 mg), SM♀L3 (AC extract of female leaves, 76 mg), SM♀L4 (water extract of female leaves, 52 mg), SM♂L1 (PE extract of male leaves, 328 mg), SM♂L2 (DE extract of male leaves, 72 mg), SM♂L3 (AC extract of male leaves, 84 mg) and SM♂L4 (water extract of male leaves, 60 mg).

### 4.3. Low-Pressure Column Chromatography

All the obtained extracts—SM♀L1, SM♀L2 and SM♂L1, SM♂L2—were fractionated by silica gel in the solid phase and solvents with increasing polarity in the mobile phase. The silica gel was packed using petroleum ether and helped by vibrations to improve the packing of the stationary phase. A layer of sand was put on top of the silica to avoid the dispersion of the solid phase and the creation of air bubbles [61]. The solvents used for the run were PE 100%, PE/Ethyl acetate (EA) 95%/5%, PE/EA 90%/10%, PE/EA 80%/20%, PE/EA 70%/30%, EA 100% and methanol 100%. The fractions were manually collected and those with a similar visible color were reunited, obtaining seven fractions from SM♀L1, four fractions from SM♀L2, nine fractions from SM♂L1 and three fractions from SM♂L2.

### 4.4. GC-MS Analysis

The active fractions were analyzed with a Perkin Elmer GC/MS Clarus 500. The gas chromatograph was equipped with a Stabilwax fused-silica capillary column (Restek, Bellafonte, PA, USA) (60m × 0.25 mm, 0.5 µm of film thickness).

The analytical conditions were set as follows: the injector was at 280 °C; the oven temperature was programmed from 60 °C to 220 °C at a rate of 5 °C/min and held for 30 min; the carrier gas was helium at 1.0 mL/min. One microliter of methanol was added to the extracts and 2 µL of each was injected directly with a split ratio of 1:20. The Clarus 500 Mass Spectrometer single quadrupole operated in the electron impact (EI) mode at 70 eV. The mass range was from 30 to 450 *m*/*z*. The relative percentages for quantification of the components were calculated by the electronic integration of the GC-FID (Gas Chromatograph-Ionization Flame Detector) peak areas using the normalization method. The identification of the components separated by GC/MS was performed by comparing the mass spectra for each compound with that reported in the MS library search (Wiley and Nist 02). Furthermore, the linear retention indices (LRI) of each compound were calculated according to van den Dool and Kratz [62], using a mixture of *n*-alkanes (C8–C30, Ultrasci) injected directly into the GC injector with the same operating conditions reported above. All the analyses were repeated twice. The percentage area values were obtained electronically from the GC-FID response without the use of an internal standard or correction factors.

### 4.5. Antibacterial Activity

The antibacterial activity was tested using selected pathogens and commensal strains from the American Type Culture Collection (ATCC) (Manassas, MD, USA): *S. aureus* ATCC 6538, *Escherichia coli* ATCC 25922, *P. aeruginosa* ATCC 9027, *E. faecalis* ATCC 29212, *B. subtilis* ATCC 6633 and *C. albicans* ATCC 10231.

The strains were maintained at 4 °C on slants of Tryptic Soy Agar (bioMerieux, Florence, Italy) and Malt Extract Agar for bacteria and molds, respectively. The bacterial and fungal strains were previously activated in Tryptic Soy Agar (TSA) at 37 °C/24 h and Sabouraud Dextrose Agar (SDA) at 25 °C/5 days.

All the inocula were prepared with fresh cultures plated the day before the test.

### 4.6. Disk Diffusion Test (DDT)

Sterile disks (6 mm diameter) impregnated with *S. molle* (L.) fractions were placed on TSA, having been previously seeded with the bacterial strain. The microorganisms were suspended in sterile Tryptic Soy broth with a turbidity from 0.5 to 1 McFarland (approximately 10^7^–10^8^ CFU/mL). *S. molle* (L.) fractions which had growth inhibitory activity were highlighted by the absence of microorganism growth around spots and the haloes were measured using a Vernier calliper rule and expressed in mm [63,64]. Each strain was tested in triplicate and the results were reported in Table 1 and Table 2. A sterilized physiological saline solution (5 μL) and dimethyl sulfoxide (DMSO) (5 μL) were used as a negative control sample.

### 4.7. Minimum Inhibitory Concentration (MIC)

The MIC values of *S. molle* (L.) fractions were determined against all strains by means of a microwell dilution method [65,66]. Dilutions of each fraction were prepared in DMSO. All 96 microplate wells were prepared by adding 95 μL of Tryptic Soy broth (TSB) and 5 μL of inoculum. Each well was inoculated with a different concentration of extracts (ranging from 1.56 to 3200 µg/mL) or fractions (ranging from 0.25 to 512 μg/mL) [67]. As a negative control, wells containing 195 μL of the nutrient broth and 5 μL of the bacterial strains without extracts were prepared. Each plate was mixed on the plate shaker at 300 rpm for 20 s and then incubated at 37 °C for 48 h. The optical density (OD) of the plates was measured at 570 nm using a microtiter plate reader. The MIC values were expressed as μg/mL, taking into account the density value of each sample. All the experiments were repeated three times [68].

### 4.8. Statistical Analysis

The results were expressed as means ± the standard deviation (SD). Data were analyzed with a one-way analysis of variance (ANOVA) using GraphPad Prism software (GraphPad Prism 5.0, GraphPad Software, Inc., San Diego, CA, USA), with *p* values of ≤ 0.05 considered statistically significant.

## 5. Conclusions

In conclusion, our findings confirm the antimicrobial properties of *S. molle* (L.) leaf extracts and derived fractions from *S. molle* (L.) male and female plants. In most cases, the obtained fractions showed improved antimicrobial activity compared to the in toto extracts.

The chemical investigations of positive fractions could suggest a possible correlation between molecules highlighted by GC-MS analysis and the antibacterial activity. Further studies are needed for a more detailed evaluation of the antibacterial properties of single identified molecules in extracts and their possible synergic effects.

## Figures and Tables

**Table 1:** Disk Diffusion Test (DDT) and Minimal Inhibitory Concentration (MIC) of *S. molle* (L.) leaf (from female plants), extracts and fractions (*p* values ≤ 0.05).

		*E. coli* ATCC 25922		*S. aureus* ATCC 6538		*P. aeruginosa* ATCC 9027		*E. faecalis* ATCC 29212		*C. albicans* ATCC 10231		*B. subtilis* ATCC 6633	
Extract	Fraction	DDT (mm)	MIC (µg/mL)	DDT (mm)	MIC (µg/mL)	DDT (mm)	MIC (µg/mL)	DDT (mm)	MIC (µg/mL)	DDT (mm)	MIC (µg/mL)	DDT (mm)	MIC (µg/mL)
SM♀L1		/	/	10.3 ± 1.5	400	/	/	/	/	16.7 ± 2.1	25	14.3 ± 1.2	400
	F1	/	/	/	/	/	/	/	/	/	/	/	/
	F2	/	/	/	/	/	/	/	/	/	/	/	/
	F3	/	/	/	/	/	/	/	/	/	/	/	/
	F4	/	/	12.3 ± 1.2	16	/	/	/	/	/	/	13.0 ± 1.7	16
	F5	/	/	/	/	/	/	/	/	/	/	24.7 ± 0.6	8
	F6	/	/	/	/	/	/	/	/	/	/	21.7 ± 1.2	8
	F7	/	/	20.0 ± 1.0	128	/	/	/	/	/	/	/	/
SM♀L2		/	/	10.3 ± 1.2	200	/	/	8.3 ± 0.6	200	14.3 ± 2.1	50	17.3 ± 1.2	400
	F1	/	/	/	/	/	/	/	/	/	/	/	/
	F2	/	/	/	/	/	/	/	/	/	/	25.7 ± 0.6	1
	F3	/	/	/	/	/	/	/	/	/	/	24.7 ± 1.2	2
	F4	/	/	17.7 ± 2.1	64	/	/	14.7 ± 1.2	128	9.7 ± 0.6	32	/	/
SM♀L3		/	/	/	/	/	/	/	/	/	/	/	/
SM♀L4		/	/	/	/	/	/	/	/	/	/	/	/

SM♀L1: petroleum ether extract; SM♀L2: diethyl ether extract; SM♀L3: acetone extract; SM♀L4: water extract.

**Table 2:** Disk Diffusion Test (DDT) and Minimal Inhibitory Concentration (MIC) of *S.molle* (L.) leaf (from male plants), extracts and fractions (*p* values ≤ 0.05).

		*E. coli* ATCC 25922		*S. aureus* ATCC 6538		*P. aeruginosa* ATCC 9027		*E. faecalis* ATCC 29212		*C. albicans* ATCC 10231		*B. subtilis* ATCC 6633	
Extract	Fraction	DDT (mm)	MIC (µg/mL)	DDT (mm)	MIC (µg/mL)	DDT (mm)	MIC (µg/mL)	DDT (mm)	MIC (µg/mL)	DDT (mm)	MIC (µg/mL)	DDT (mm)	MIC (µg/mL)
SM♂L1		/	/	8.7 ± 0.6	400	/	/	/	/	15.3 ± 1.5	400	13.7 ± 0.6	25
	F1	/	/	13.3 ± 1.2	128	/	/	/	/	/	/	26.3 ± 0.6	128
	F2	/	/	/	/	/	/	/	/	/	/	12.7 ± 2.1	256
	F3	/	/	/	/	/	/	/	/	/	/	16.3 ± 2.9	>512
	F4	/	/	/	/	/	/	/	/	/	/	/	/
	F5	/	/	/	/	/	/	/	/	/	/	12.7 ± 0.6	512
	F6	/	/	16.7 ± 0.6	8	/	/	/	/	/	/	/	/
	F7	/	/	15.3 ± 0.6	16	/	/	/	/	/	/	/	/
	F8	/	/	/	/	/	/	/	/	/	/	/	/
	F9	/	/	/	/	/	/	/	/	/	/	/	/
	F10	/	/	15.3 ± 1.2	128	/	/	/	/	/	/	/	/
SM♂L2		/	/	/	/	/	/	/	/	12.7 ± 0.6	400	11.3 ± 1.5	100
	F1	/	/	/	/	/	/	/	/	8.0 ± 1.0	256	/	/
	F2	/	/	/	/	/	/	/	/	6.0 ± 1.7	128	/	/
	F3	/	/	/	/	/	/	/	/	5.0 ± 1.0	128	/	/
	F4	/	/	/	/	/	/	/	/	10.3 ± 0.6	128	/	/
	F5	/	/	/	/	/	/	/	/	/	/	/	/
	F6	/	/	/	/	/	/	/	/	11.7 ± 1.5	64	28.3 ± 1.2	16
	F7	/	/	/	/	/	/	/	/	/	/	29.7 ± 0.6	256
SM♂L3		/	/	/	/	/	/	/	/	/	/	/	/
SM♂L4		/	/	/	/	/	/	/	/	/	/	/	/

SM♂L1: petroleum ether extract; SM♂L2: diethyl ether extract. SM♂L3: acetone extract; SM♂L4: water extract.

**Table 3:** Chemical composition (%) of petroleum ether and diethyl ether fractions from female *S. molle* (L.) leaves.

	Components ^1^	LRI ^2^	LRI ^lit 3^	SM♀L1F4	SM♀L1F5	SM♀L1F6	SM♀L1F7	SM♀L2F2	SM♀L2F3	SM♀L2F4
1	β-terpinene	1200	1206	33.8	-	-	-	5.8	-	-
2	cyclotetradecene	1680	*	-	-	-	15.7	-	-	77.6
3	Elemol	2093	2090	38.1	74.6	71.6	2.7	-	-	-
4	viridiflorol	2092	2091	-	3.6	-	-	-	-	-
5	3,7,11,15-tetramethyl-2-hexadecen-1-ol	2125	*	-	-	-	-	86.9	-	-
6	dehydroxyisocalamendiol	2141	*	8.5	-	2.9	3.4	-	-	-
7	γ-eudesmol	2191	2185	5.2	4.3	4.2	6.4	1.0	-	-
8	α-eudesmol	2240	2232	4.1	9.4	3.9	3.9	1.0	-	-
9	β-eudesmol	2258	2249	10.2	8.0	17.4	67.9	1.3	-	-
10	2,4-di-tert-butyl-phenol	2320	2321	-	-	-	-	-	-	22.4
11	Phytol	2625	2633	-	-	-	-	-	100.0	-
12	hexadecanoic acid	2880	2887	-	-	-	-	4.0	-	-
SUM				99.9	99.9	100.0	100.0	100.0	100.0	100.0
Monoterpene hydrocarbons				33.8				5.8		
Sesquiterpene hydrocarbons				57.6	99.9	97.1	80.9	3.3		
Oxygenated sesquiterpenes				8.5		2.9	3.4			
Diterpenoid alcohol								86.9	100.0	
Others							15.7	4.0		100.0

^1^ elution order on polar column; ^2^ linear retention indices (LRI) measured on polar column; ^3^ linear retention indices from literature; * LRI ^lit^ not available; (SM♀L1F4–L1F7): fractions from petroleum ether female extracts; (SM♀L2F2–L2F4): fractions from diethyl ether female extracts.

**Table 4:** Chemical composition (%) of petroleum ether and diethyl ether fractions from male *S. molle* (L.) leaves.

	Components ^1^	LRI ^2^	LRI ^lit 3^	SM♂L1F1	SM♂L1F2	SM♂L1F3	SM♂L1F5	SM♂L1F6	SM♂L1F7	SM♂L1 F10	SM♂L2F4	SM♂L2F6	SM♂L2F7
1	α-pinene	1033	1035	-	-	-	0.7	-	-	-	-	-	-
2	2-propanone, 1-hydroxy	1315	1317	-	-	-	-	-	-	-	-	-	1.9
3	isoledene	1340	*	0.4	-	-	-	-	-	-	-	-	-
4	isoshybunone	1480	1482	-	-	85.0	-	-	-	-	-	-	-
5	Elixene	1510	1514	9.9	9.2	-	-	-	-	-	-	-	-
6	α-gurjunene	1556	1549	2.3	0.2	-	-	-	-	-	-	-	-
7	β-ylangene	1570	1574	1.0	0.4	-	-	-	-	-	-	-	-
8	β-elemene	1608	1598	2.4	5.3	-	-	-	-	-	-	-	-
9	β-copaene	1635	1631	0.6	-	-	-	-	-	-	-	-	-
10	β-caryophyllene	1637	1634	7.4	0.8	-	-	-	-	-	-	-	-
11	cis-β-terpineol	1640	1644	-	-	-	1.9	-	-	-	-	-	-
12	γ-elemene	1648	1650	0.2	0.5	-	-	-	-	-	-	-	-
13	aromadendrene	1675	1670	2.0	0.4	-	-	-	-	-	-	-	-
14	cyclotetradecane	1680	*	-	-	-	-	-	-	-	-	-	8.9
16	oxalic acid	1691	*	-	-	-	-	-	-	-	-	-	18.5
15	humulene	1700	1693	2.2	4.5	-	-	-	-	-	-	-	-
17	Ledene	1698	1695	5.8	14.1	-	-	-	-	-	-	-	-
18	germacrene D	1728	1726	51.8	5.6	-	-	-	-	-	-	-	-
19	α-muurolene	1730	1729	-	20.2	-	-	-	-	-	-	-	-
20	δ-cadinene	1758	1758	10.3	24.3	-	-	-	-	-	-	-	-
21	γ-cadinene	1778	1782	2.3	5.9	-	-	-	-	-	-	-	-
22	isoaromadendrene epoxide	1800	1807	-	-	-	-	-	7.0	8.3	-	-	-
23	nonadecene	1932	1927	-	-	-	-	-	-	-	-	-	28.8
24	1-docosene	2000	*	-	-	-	-	-	-	-	-	-	11.8
25	Elemol	2091	2090	-	-	-	3.2	-	-	-	4.2	-	-
26	viridiflorol	2092	2091	-	-	-	2.0	-	-	-	3.6	-	-
27	3,7,11,15-tetramethyl-2-hexadecen-1-ol	2125	*	-	-	-	16.9	-	-	-	-	-	-
28	spathulenol	2142	2136	0.2	3.8	-	-	2.3	14.2	-	-	-	-
29	γ-eudesmol	2178	2180	-	-	-	-	-	-	-	6.2	-	-
30	α-eudesmol	2240	2232	-	-	-	12.0	-	-	-	26.9	-	-
31	dehydroxyisocalamendiol	2242	*	-	-	11.7	-	-	-	-	18.8	-	-
32	hexadecanoic acid, methyl ester	2244	2233	-	-	2.4	-	-	-	-	-	-	-
33	hexadecanoic acid, ethyl ester	2247	2246	-	-	0.8	-	-	-	-	-	-	-
34	β-eudesmol	2255	2249	-	-	-	57.6	12.9	59.6	91.7	40.1	-	8.8
35	phenol 2,4-di-tert-butyl-	2322	2321	-	-	-	-	-	-	-	-	-	7.3
36	cyclopropanetetradecanoic acid, 2-octyl-,methyl ester	2405	*	-	-	-	-	-	-	-	-	-	14.0
37	isocalamendiol	2510	2500	1.1	4.4	-	-	84.8	12.2	-	-	-	-
38	benzyl benzoate	2650	2652	-	-	-	5.6	-	-	-	-	-	-
39	squalene	2860	2865	-	-	-	-	-	7.0	-	-	-	-
40	hexadecanoic acid	2880	2887	-	-	-	-	-	-	-	-	100.0	-
SUM				99.9	99.6	99.9	99.9	100.0	100.0	100.0	99.8	100.0	100.0
Monoterpene hydrocarbons				51.8	5.6		0.7						
Monoterpenes alcohol							1.9						
Sesquiterpene hydrocarbons				40.1	89.4	85.0	74.8	97.7	71.8	91.7	81.0		8.8
Oxygenated sesquiterpenes						11.7					18.8		
Tricyclic sesquiterpenes				0.2	3.8			2.3	14.2				
Triterpenes									7.0				
Bicyclic sesquiterpenes				7.4	0.8								
Others				0.4		3.2	22.5		7.0	8.3		100.0	91.2

^1^ elution order on polar column; ^2^ linear retention indices measured on polar column; ^3^ linear retention indices from literature; * LRI ^lit^ not available; (SM♂L1F1–L1F10): fractions from petroleum ether male extracts; (SM♂L2F4–L2F7): fractions from diethyl ether male extracts.

## References

[1] Duval R.E., Grare M., Demoré B. (2019). Fight against antimicrobial resistance: We always need new antibacterials but for right bacteria. Molecules.

[2] Davies J., Davies D. (2010). Origins and evolution of antibiotic resistance. Microbiol. Mol. Biol. Rev..

[3] De Gomes R.B.A., de Souza E.S., Gerhardt Barraqui N.S., Tosta C.L., Nunes A.P.F., Schuenck R.P., Ruas F.G., Ventura J.A., Filgueiras P.R., Kuster R.M. (2019). Residues from the Brazilian pepper tree (*Schinus terebinthifolia* Raddi) processing industry: Chemical profile and antimicrobial activity of extracts against hospital bacteria. Ind. Crops Prod..

[4] Sharma K., Mahato N., Lee Y.R. (2018). Systematic study on active compounds as antibacterial and antibiofilm agent in aging onions. J. Food Drug Anal..

[5] Gundidza M. (1993). Antimicrobial activity of essential oil from *Schinus molle* Linn. Cent. Afr. J. Med..

[6] Belhamel K., Abderrahim A., Ludwig R. (2008). Chemical composition and antibacterial activity of the essential oil of *Schinus molle* L. grown in Algeria. Int. J. Essent. Oil Ther..

[7] El Hayouni A., Chraief I., Abedrabba M., Bouix M., Leveau J.Y., Mohammed H., Hamdi M. (2008). Tunisian *Salviaofficinalis* L. and *Schinus mole* L. essential oils: Their chemical compositions and their preservative effects against *Salmonella* inoculated in minced beef meat. Int. J. Food Microbiol..

[8] Rocha P.M.D.M., Rodilla J.M., Díez D., Elder H. (2012). Synergistic antibacterial activity of the essential oil of aguaribay (*Schinus mole* L.). Molecules.

[9] Martins M.D.R., Arantes S., Candeias F., Tinoco M.T., Cruz-Morais J. (2014). Antioxidant, antimicrobial and toxicological properties of *Schinus molle* L. Essential Oils. J. Ethnopharmacol..

[10] Iseppi R., Brighenti V., Licata M., Lambertini A., Sabia C., Messi P., Pellati F., Benvenuti S. (2019). Chemical characterization and evaluation of the antibacterial activity of essential oils from fibre-type *Cannabis sativa L.* (Hemp). Molecules.

[11] Garzoli S., Turchetti G., Giacomello P., Tiezzi A., Laghezza Masci V., Ovidi E. (2019). Liquid and vapour phase of lavandin (*Lavandula × intermedia*) essential oil: Chemical composition and antimicrobial activity. Molecules.

[12] Lim T.K. (2012). Edible medicinal and non-medicinal plants. Edible Med. Non-Med. Plants.

[13] Diaz C., Quesada S., Brenes O., Aguilar G., Ciccio J.F. (2008). Chemical composition of *Schinus molle* essential oil and its cytotoxic activity on tumour cell lines. Nat. Prod. Res..

[14] Bendaoud H., Romdhane M., Souchard J.P., Cazaux S., Bouajila J. (2010). Chemical composition and anticancer and antioxidant activities of *Schinus mole* L. and *Schinus terebinthifolius* Raddi berries essential oils. J. Food Sci..

[15] Aboalhaija N.H., Awwad O., Khalil E., Abbassi R., Abaza I.F., Afifi F.U., Al R., Street A., Rania Q., Abdallah A. (2019). Chemodiversity and antiproliferative activity of the essential oil of *Schinusmolle* growing in Jordan. Chem. Biodivers..

[16] Ovidi E., Garzoli S., Laghezza Masci V., Turchetti G., Tiezzi A. (2019). GC-MS investigation and antiproliferative activities of extracts from male and female flowers of *Schinus molle* L. Nat. Prod. Res..

[17] Ibrahim B., Al-Naser Z. (2014). Analysis of fruits *Schinus molle* extractions and the efficacy in inhibition of growth the fungi in laboratory. Int. J. Chem. Tech. Res..

[18] Prado A.C., Garces H.G., Bagagli E. (2018). *Schinusmolle* essential oil as a potential source of bioactive compounds: Antifungal and antibacterial properties. J. Appl. Microbiol..

[19] Belle R., Barrachina M.D., Moreno L., Esplugues J. (1996). Effects on arterial blood pressure of the methanol and dichloromethanol extracts from *Schinus mole* L. in Rats. Phyther. Res..

[20] Yueqin Z., Recio M.C., Máñez S., Giner R.M., Cerdá-Nicolás M., Ríos J.L. (2003). Isolation of two triterpenoids and a biflavanone with anti-inflammatory activity from *Schinus molle* fruits. Planta Med..

[21] Barrachina M.D., Bello R., Martínez-Cuesta M.A., Primo-Yúfera E., Esplunges J. (1997). Analgesic and central depressor effects of the dichloromethanol extract from *Schinus mole* L. Phyther. Res..

[22] Habte G. (2018). Antimalarial Activity of Aqueous and 80% Methanol Crude Seed Extracts and Solvent Fractions of *Schinus mole Linnaeus* (*Anacardiaceae*) in *Plasmodium berghei* Infected Mice. Ph.D. Thesis.

[23] Feyera T., Abdisa E. (2016). In vitro acaricidal activity of crude extracts of *Schinus molle* (L.) leaves against field population of *Bophilus decoloratus* and *Rhipicephaluspulchellus* ticks. Afr. J. Pharm. Pharmacol..

[24] Rey-Valeirón C., Pérez K., Guzmán L., López-Vargas J., Valarezo E. (2018). Acaricidal effect of *Schinusmolle* (*Anacardiaceae*) essential oil on unengorged larvae and engorged adult females of *Rhipicephaluss anguineus* (Acari: *Ixodidae*). Exp. App. Acarol..

[25] Ferrero A., Werdin González J.O., Sánchez Chopa C. (2006). Biological activity of *Schinus molle* on *Triatoma infestans*. Fitoterapia.

[26] Ferrero A., Minetti A., Bras C., Zanetti N. (2007). Acute and subacute toxicity evaluation of ethanolic extract from fruits of *Schinus molle* in rats. J. Ethnopharmacol..

[27] Machado C.D., Raman V., Rehman J.U., Maia B.H.L.N.S., Meneghetti E.K., Almeida V.P., Silva R.Z., Farago P.V., Khan I.A., Budel J.M. (2019). *Schinus molle*: Anatomy of leaves and stems, chemical composition and insecticidal activities of volatile oil against bed bug (*Cimexlectularius*). Rev. Bras. Farmacogn..

[28] Deveci O., Sukan A., Tuzun N., Kocabas E.E.H. (2010). Chemical composition, repellent and antimicrobial activity of *Schinus molle* L. J. Med. Plants Res..

[29] Pérez-López A., Cirio A.T., Rivas-Galindo V.M., Aranda R.S., de Torres N.W. (2011). Activity against *Streptococcus pneumoniae* of the essential oil and δ-cadinene isolated from *Schinus molle* fruit. J. Essent. Oil Res..

[30] Mehani M., Segni L. (2013). Antimicrobial effect of essential oil of plant *Schinus molle* on some bacteria pathogens. World Acad. Sci. Eng. Technol. Int. J. Chem. Nucl. Metall. Mater. Eng..

[31] Salem M.Z., Zayed M.Z., Ali H.M., El-Kareem M.S.A. (2016). Chemical composition, antioxidant and antibacterial activities of extracts from *Schinus molle* wood branch growing in Egypt. J. Wood Sci..

[32] Eryigit T., Yildirim B., Ekici K., Çirka M. (2017). Chemical composition, antimicrobial and antioxidant properties of *Schinus molle* L. Essential oil from Turkey. J. Essent. Oil Bear. Plants.

[33] Garzoli S., Laghezza Masci V., Turchetti G., Pesci L., Tiezzi A., Ovidi E. (2018). Chemical investigations of male and female leaf extracts from *Schinus molle* L. Nat. Prod. Res..

[34] Hashem F.A., Saleh M.M. (1999). Antimicrobial components of some cruciferae plants (*Diplotaxis harra* Forsk. and *Erucaria microcarpa* Boiss.). Phyther. Res..

[35] Yff B.T.S., Lindsey K.L., Taylor M.B., Erasmus D.G., Jäger A.K. (2002). The pharmacological screening of *Pentanisia prunelloides* and the isolation of the antibacterial compound palmitic acid. J. Ethnopharmacol..

[36] Costa E.V., Teixeira S.D., Marques F.A., Duarte M.C.T., Delarmelina C., Pinheiro M.L.B., Trigo J.R., Sales Maia B.H.L.N. (2008). Chemical composition and antimicrobial activity of the essential oils of the Amazon *Guatteriopsis* species. Phytochemistry.

[37] Rodrigues F.G., Rodrigues F.G., Almeida S.X., Campos A., Oliveira L.S., Saraiva M., Cabral M.S., Costa J.G. (2012). Chemical composition, antibacterial and antifungal activities of essential oil from *Cordiaverbenacea* DC leaves. Pharmacogn. Res..

[38] Zeraib A., Ramdani M., Boudjedjou L., Chalard P., Figuredo G. (2014). Chemical composition and antibacterial activity of *Juniperus thurifera* L. essential oils. J. BioSci. Biotech..

[39] Dahham S.S., Tabana Y.M., Iqbal M.A., Ahamed M.B.K., Ezzat M.O., Majid A.S.A., Majid A.M.S.A. (2015). The anticancer, antioxidant and antimicrobial properties of the sesquiterpene β-caryophyllene from the essential oil of *Aquilaria crassna*. Molecules.

[40] Mohammed G.J., Omran A.M., Hussein H.M. (2016). Antibacterial and phytochemical analysis of *Piper nigrum* using gas chromatography—Mass spectrum and fourier-transform infrared spectroscopy. Int. J. Pharmacogn. Phytochem. Res..

[41] Bajalan I., Rouzbahani R., Pirbalouti A.G., Maggi F. (2017). Variation in chemical composition and antibacterial activity of the essential oil of wild populations of *Phlomis olivieri*. Chem. Biodivers..

[42] Montanari R.M., Barbosa L.C.A., Demuner A.J., Silva C.J. (2017). Chemical composition and antibacterial activity of essential oil from *Verbenaceae* species: Alternative sources of (E)-caryophyllene and Germacrene-D. Quim. Nov..

[43] El Kamari F.T.A., El Atki Y., Aouam I., Oumokhtar B., Lyoussi B.A.A. (2018). *Cymbopogon Nardus,* L. Essential Oil: Phytochemical screening and its antibacterial activity against clinical bacteria responsible for nosocomial infections in neonatal intensive care. Int. J. Pharm. Sci. Rev. Res..

[44] Sieniawska E., Sawicki R., Golus J., Swatko-Ossor M., Ginalska G., Skalicka-Wozniak K. (2018). *Nigella damascena,* L. essential oil-a valuable source of β-elemene for antimicrobial testing. Molecules.

[45] Wang H., Liu Y. (2010). Chemical composition and antibacterial activity of essential oils from different parts of *Litsea cubeba*. Chem. Biodivers..

[46] Padmavathi A.R., Bakkiyaraj D., Thajuddin N., Pandian S.K. (2015). Effect of 2, 4-di-tert-butylphenol on growth and biofilm formation by an opportunistic fungus *Candida albicans*. Biofouling.

[47] Varsha K.K., Devendra L., Shilpa G., Priya S., Pandey A., Nampoothiri K.M. (2015). 2,4-di-tert-butyl phenol as the antifungal, antioxidant bioactive purified from a newly isolated *Lactococcus* sp. Int. J. Food Microbiol..

[48] Dr. Duke’s Phytochemical and Ethnobotanical Databases. https://data.nal.usda.gov/dataset/dr-dukes-phytochemical-and-ethnobotanical-databases.

[49] Aissaoui N., Mahjoubi M., Nas F., Mghirbi O., Arab M., Souissi Y., Hoceini A., Masmoudi A.S., Mosbah A., Cherif A. (2019). Antibacterial potential of 2,4-di-tert-butylphenol and calixarene-based prodrugs from thermophilic *Bacillus licheniformis* isolated in Algerian hot spring. Geomicrobiol. J..

[50] Al-qudah M.A., Muhaidat R., Trawenh I.N., Al Jaber H.I., Zarga M.H.A. (2012). Volatile constituents of leaves and bulbs of *Gynandriris sysyrinchium* and their antimicrobial activities. Jordan J. Chem..

[51] Islam M.T., Ali E.S., Uddin S.J., Shaw S., Islam M.A., Ahmed M.I., Billah M.M. (2018). Phytol: A review of biomedical activities. Food Chemical. Toxicol..

[52] Pellegrini C., Alonso-Salces R.M., Umpierrez L., Rossini C. (2017). Chemical composition, antimicrobial activity, and mode of action of essential oils against *Paenibacillus larvae*, etiological agent of American foulbrood on *Apis mellifera*. Chem. Biodivers..

[53] Labed-Zouad I., Labed A., Laggoune S., Zahia S., Kabouche A., Kabouche Z. (2015). Chemical compositions and antibacterial activity of four essential oils from *Ferula vesceritensis* Coss. & Dur. against clinical isolated and food-borne pathogens. Rec. Nat. Prod..

[54] Meratate F., Lalaoui A., Rebbas K., Belhadad O.K., Hammadou N.I., Meratate H., Demirtas I., Akkal S., Laouer H. (2016). Chemical composition of the essential oil of *Carduncellus helenioides* (Desf.) Hanelt from Algeria. Orient. J. Chem..

[55] Kadhim M.J., Mohammed G.J., Hameed I.H. (2016). In vitro antibacterial, antifungal and phytochemical analysis of methanolic extract of fruit *Cassia fistula*. Orient. J. Chem..

[56] Sharma R., Kishore N., Hussein A., Lall N. (2013). Antibacterial and anti-inflammatory effects of *Syzygiumjambos* L. (Alston) and isolated compounds on acne vulgaris. BMC Complement. Altern. Med..

[57] Rahuman A.A., Gopalakrishnan G., Ghouse S., Arumugam S., Himalayan B. (2000). Effect of Feronia limonia on mosquito larvae. Fitoterapia.

[58] Aparna V., Dileep K.V., Mandal P.K., Karthe P., Sadasivan C., Haridas M. (2012). Anti-inflammatory property of n-hexadecanoic acid: Structural evidence and kinetic assessment. Chem. Biol. Drug Des..

[59] Kwak A.M., Lee I.K., Lee S.Y., Yun B.S., Kang H.W. (2016). Oxalic acid from *Lentinula edodes* culture filtrate: Antimicrobial activity on phytopathogenic bacteria and qualitative and quantitative analyses. Mycobiology.

[60] Zeng Q.Y., Wu J., Lin P.C. (2016). Chemical composition and antimicrobial activity of the essential oil from *Epilobium Angustifolium*. Chem. Nat. Compd..

[61] Garzoli S., Masci V.L., Ovidi E., Turchetti G., Zago D., Tiezzi A. (2019). Chemical investigation of a biologically active *Schinus Molle* L. leaf extract. J. Anal. Methods Chem..

[62] Van Den Dool H., Kratz P.D. (1963). A generalization of the retention index system including linear temperature programmed gas—Liquid partition chromatography. J. Chromatogr. A.

[63] Klancnik A., Piskernik S., Jersek B., Mozina S.S. (2010). Evaluation of diffusion and dilution methods to determine the antibacterial activity of plant extracts. J. Microbiol. Methods.

[64] CLSI (2016). Performance Standards for Antimicrobial Susceptibility Testing.

[65] CI (2016). Performance Standards for Antimicrobial Disk.

[66] Sahin F., Güllüce M., Daferera D., Sökmen A., Sökmen M., Polissiou M., Agar G., Özer H. (2004). Biological activities of the essential oils and methanol extract of *Origanum vulgare* ssp. *vulgare* in the Eastern Anatolia region of Turkey. Food Control.

[67] Saeed S., Tariq P. (2008). In vitro antibacterial activity of clove against Gram negative bacteria. Pak. J. Bot..

[68] Pinto E., Vale-Silva L., Cavaleiro C., Salgueiro L. (2009). Antifungal activity of the clove essential oil from *Syzygium aromaticum* on *Candida*, *Aspergillus* and dermatophyte species. J. Med. Microbiol..

